# Role of Noncontrast Cone-Beam Computed Tomography in Pulmonary Arteriovenous Malformation Embolization

**DOI:** 10.3390/jcm15145688

**Published:** 2026-07-20

**Authors:** Juil Park, Joon Ho Kwon, Man-Deuk Kim, Jong Yun Won, Gyoung Min Kim, Jaesung Cho, Kichang Han, Seokmin Jeong

**Affiliations:** 1 Department of Radiology, Severance Hospital, Research Institute of Radiological Science, Yonsei University College of Medicine, 50-1, Yonsei-Ro, Seodaemun-gu, Seoul 03722, Republic of Korea; wnolf2@gmail.com (J.P.); mdkim@yuhs.ac (M.-D.K.); jywon@yuhs.ac (J.Y.W.); gyoungmin@yuhs.ac (G.M.K.); jaesungjo@yuhs.ac (J.C.); 2 Division of Interventional Radiology, Department of Radiology, UCLA Medical Center, David Geffen School of Medicine at UCLA, 757 Westwood Plaza, Los Angeles, CA 90095, USA; kihan@mednet.ucla.edu; 3 Department of Radiology, Chung-Ang University Hospital, Chung-Ang College of Medicine, 102, Heukseok-ro, Dongjak-gu, Seoul 06973, Republic of Korea; fofrl@naver.com

**Keywords:** pulmonary arteriovenous malformation, embolization, cone-beam computed tomography

## Abstract

**Objective**: To evaluate the role of noncontrast cone-beam computed tomography (CBCT) in pulmonary arteriovenous malformation (PAVM) embolization. **Materials and Methods**: This retrospective single-center study included 63 patients (mean age, 51.3 ± 12.1 years; 8 males, 55 females) who underwent PAVM embolization between January 2018 and June 2024. Patients were categorized into a CBCT-assisted group (*n* = 13) and a digital subtraction angiography (DSA)-only group (*n* = 50). The use of CBCT was determined selectively at the discretion of the performing interventional radiologists. Patient demographics, PAVM characteristics, radiation exposure, fluoroscopy time, procedure time, and the number of DSA acquisitions were compared between the two groups. Technical and clinical success were evaluated. **Results**: CBCT guidance significantly reduced the number of DSA acquisitions (2.4 ± 1.0 vs. 4.9 ± 2.6, *p* = 0.001), procedure time (54.6 ± 24.3 min vs. 75.1 ± 34.5 min, *p* = 0.021), and fluoroscopy time (14.9 ± 12.0 min vs. 25.5 ± 18.2 min, *p* = 0.017) compared with DSA-only group. Dose-area product (87.7 ± 111.4 Gy·cm^2^ vs. 86.0 ± 58.7 Gy·cm^2^; *p* = 0.959) and cumulative air-kerma (387.2 ± 464.2 mGy vs. 354.5 ± 305.7 mGy; *p* = 0.811) did not differ significantly between the groups. Technical success was achieved in all cases (100%). Clinical success rates were 98% in the DSA-only group and 100% in the CBCT group, with no significant difference (*p* = 1.000). No procedure-related complications were observed. **Conclusions**: Noncontrast CBCT may improve procedural efficiency in PAVM embolization by reducing DSA acquisitions and procedure time without significantly increasing radiation dose. This technique provides valuable anatomical guidance, particularly in complex scenarios such as obscured or multiple lesions.

## 1. Introduction

Pulmonary arteriovenous malformations (PAVMs) are rare vascular anomalies characterized by direct connections between the pulmonary artery and vein, bypassing the capillary bed and resulting in right-to-left shunts [1,2,3,4]. While often asymptomatic, they pose serious risks of paradoxical embolism, brain abscess, stroke, or life-threatening hemorrhage [5,6]. Transcatheter embolization is the established standard of care, offering high technical success with a low incidence of adverse events [7,8,9]. However, precise catheter navigation can be challenging on conventional two-dimensional (2D) digital subtraction angiography (DSA), particularly when feeding arteries are obscured by overlapping vascular structures, the cardiac silhouette, or the diaphragm [10]. Such anatomical complexities often necessitate multiple angiographic acquisitions in various projections, potentially increasing procedure time, and radiation exposure.

Cone-beam computed tomography (CBCT) has emerged as a vital adjunct in interventional radiology by providing high-resolution 3D anatomical data and real-time fluoroscopic overlays [11,12,13,14,15,16,17,18]. While previously reported CBCT protocols for pulmonary artery interventions—primarily for chronic thromboembolic pulmonary hypertension—typically require contrast-enhanced acquisitions [19,20], the approach evaluated in this study utilizes noncontrast CBCT for 3D vascular mapping in patients with PAVM. From noncontrast CBCT data, a 3D vascular roadmap is provided as a fluoroscopic overlay. This technique facilitates precise feeder identification and navigation during the mapping phase, potentially streamlining the procedural workflow by bypassing the iterative trial-and-error process associated with traditional 2D imaging.

Therefore, this study aimed to evaluate the impact of noncontrast CBCT protocol on procedural outcomes, specifically focusing on efficiency and radiation exposure, during PAVM embolization.

## 2. Materials and Methods

### 2.1. Patients

**Figure 1:**
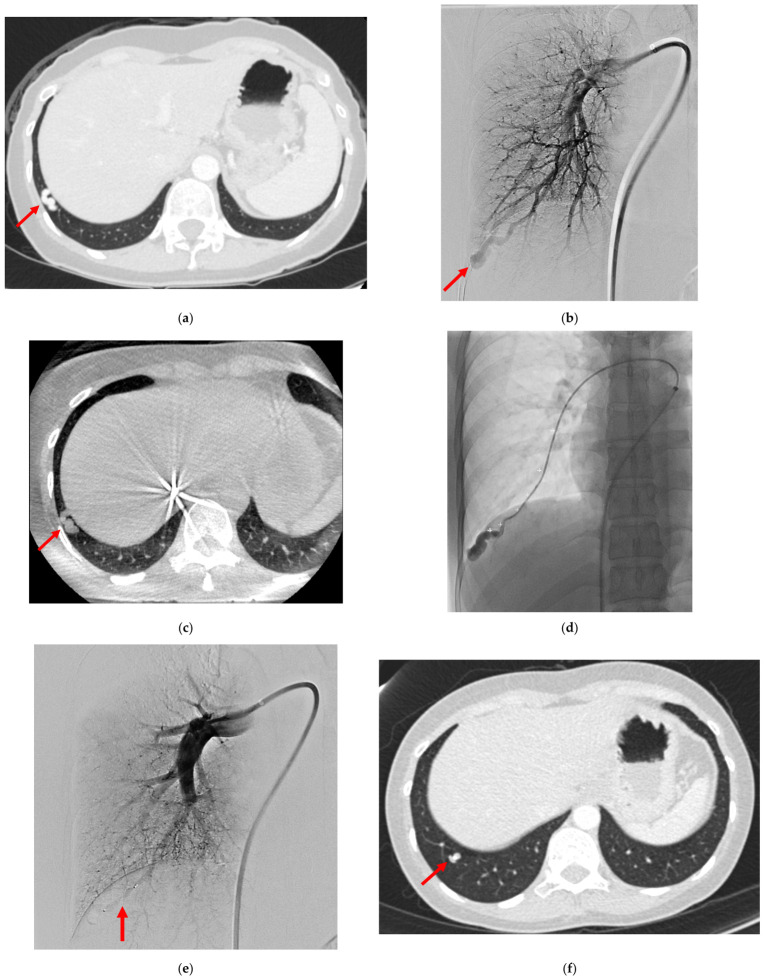
A 37-year-old woman with a pulmonary arteriovenous malformation (PAVM). (**a**) Preprocedural CT in the lung window shows the nidus of the PAVM (arrow) in the right lower lobe. (**b**) Digital subtraction angiography (DSA) from the right main pulmonary artery demonstrates the target PAVM (arrow). (**c**) Noncontrast cone-beam CT (CBCT) depicts the PAVM (arrow) in the same location as on preprocedural CT. (**d**) Overlay fluoroscopy after CBCT shows the trajectory of the feeding artery from the right main pulmonary artery to the nidus; note how the catheter closely follows the CBCT-derived trajectory. (**e**) Completion DSA confirms successful embolization with a vascular plug (arrow) and absence of opacification in the draining vein. (**f**) Follow-up CT obtained at 12 months later demonstrates complete resolution of the PAVM with the vascular plug (arrow) remaining in situ.

**Figure 2:**
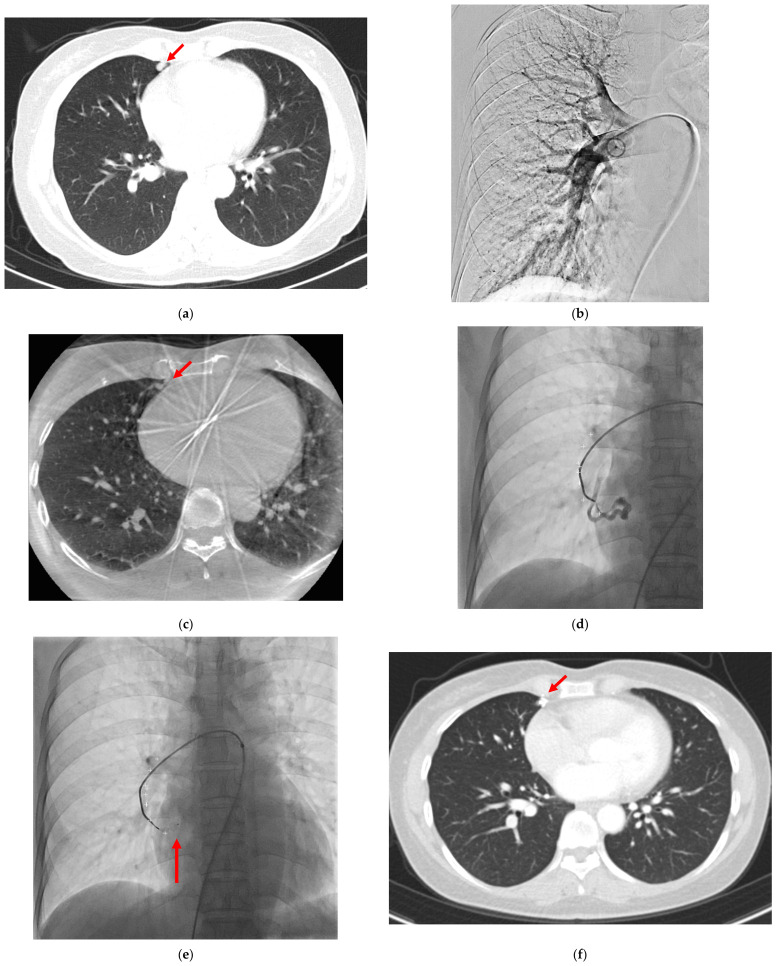
A 50-year-old woman with a pulmonary arteriovenous malformation (PAVM). (**a**) Preprocedural CT in the lung window reveals the nidus of the PAVM (arrow) in the right middle lobe; the lesion is obscured by the adjacent cardiac silhouette. (**b**) Digital subtraction angiography (DSA) from the right main pulmonary artery fails to clearly delineate the PAVM due to overlap with the heart shadow. (**c**) Noncontrast cone-beam CT (CBCT) localizes the PAVM (arrow) in the same position as on preprocedural CT. (**d**) Overlay fluoroscopy after CBCT demonstrates the course of the feeding artery from the right main pulmonary artery to the nidus. With the catheter advanced along this CBCT-derived trajectory, contrast injection clearly opacifies the PAVM. (**e**) Deployment of a vascular plug (arrow) achieves successful embolization. (**f**) Three-month follow-up CT confirms complete resolution of the PAVM with the vascular plug (arrow) in place.

This single-center retrospective study was approved by the Institutional Review Board (IRB: 4-2025-1019), and the requirement for informed consent was waived. Between January 2018 and June 2024, a total of 153 patients who underwent PAVM embolization were reviewed. Patients lacking radiation dose reports or follow-up computed tomography (CT) were excluded, leaving 63 patients (51.3 ± 12.1 years; 8 males and 55 females) for analysis. The cohort was categorized into two groups: the CBCT group and the digital subtraction angiography (DSA)-only group. CBCT was performed at the operator’s discretion, based on clinical judgment and technical considerations such as suspected lesion overlap. Data on patient demographics and PAVM characteristics—including the number, anatomical location, multiplicity, and the sizes of the feeding artery and sac—were collected (Figure 1a and Figure 2a). Obscuration was defined as cases where the feeding artery of the target PAVM was obscured by the mediastinum, diaphragm or adjacent pulmonary branches during DSA at the main pulmonary artery, resulting in poor visualization of the feeding artery.

### 2.2. Embolization Technique

All procedures were performed on an inpatient basis. Embolization was carried out in an angiography suite equipped with a flat-panel C-arm CT system (Artis Zee; Siemens Healthineers, Erlangen, Germany) by two interventional radiologists with 12 and 10 years of experience.

After local anesthesia with 2% lidocaine (Daihan Pharmaceutical Co., Ltd., Seoul, Republic of Korea), the right common femoral vein was accessed under ultrasound guidance (LOGIQ E9; GE Healthcare, Chicago, IL, USA), and a 6 Fr guiding sheath (Cook Medical, Bloomington, IN, USA) was introduced. Right or left pulmonary artery angiography was then performed using a 5-Fr pigtail catheter (Cook Medical, Bloomington, IN, USA) or the guiding sheath to localize the PAVM and identify the feeding arteries (Figure 1b and Figure 2b). In the DSA-only group, when the feeding arteries were not clearly visualized on angiography, additional angiograms were obtained at different tube angles to adequately identify the feeding arteries prior to selective catheterization.

In the CBCT group, noncontrast CBCT was performed. Imaging was acquired during a single breath-hold of 6 s with a 0.5° rotational increment, using a 512 × 512 projection matrix over a total angle of 200° at approximately 33° per second. The acquisition consisted of 419 projections, with a reference air-kerma of approximately 250 μGy per frame, obtained on the angiography system (Figure 1c and Figure 2c). The acquired volumetric dataset was subsequently transferred to a dedicated workstation (Siemens Healthineers) for three-dimensional reconstruction. Noncontrast CBCT images were then carefully compared with the preprocedural CT, which provided key anatomical landmarks. By step-by-step correlation with the preprocedural CT, the trajectory of the feeding artery was traced, allowing for differentiation of the target feeding artery from adjacent vascular structures despite the inherent challenge of distinguishing arteries and veins on noncontrast CBCT. If the vessel’s identity was unclear (i.e., whether it was a pulmonary artery or vein), the vessel could be traced along its entire length to determine if it was connected to the main pulmonary artery trunk or the left atrium. The entire trajectory from the main pulmonary artery to the nidus of the PAVM was marked, and the fluoroscopic overlay of the CBCT was used to project this path onto live fluoroscopy, guiding catheter navigation (Figure 1d and Figure 2d).

Once the feeding arteries were located, a 5 Fr catheter or a coaxial 2.0 Fr microcatheter (Progreat; Terumo, Tokyo, Japan) was advanced as close to the nidus as possible for selective embolization. Contrast agent was then injected to confirm the arteriovenous malformation nidus, followed by embolization using a vascular plug (Abbott Laboratories, Chicago, IL, USA) or detachable microcoils (Concerto; Medtronic, Minneapolis, MN, USA or Interlock; Boston Scientific, Marlborough, MA, USA) (Figure 1e and Figure 2e). The type, size, and number of embolic materials were determined by the type of delivery catheter, the diameter of the feeding artery, and the operator’s preference. After embolization, completion angiography was performed 5 min later to ensure complete vessel occlusion.

### 2.3. Technical and Clinical Outcomes

Radiation exposure metrics, including dose–area product (DAP, Gy·cm^2^; also referred to as air kerma-area product [PKA] per International Commission on Radiation Units and Measurements Report 74 and International Atomic Energy Agency Technical Reports Series No. 457 nomenclature) and cumulative air-kerma (mGy), fluoroscopy time, procedure time, and the number of DSA acquisitions, were recorded. In the CBCT-assisted group, these metrics included the radiation dose from the CBCT acquisition. Procedure time was defined as the period between the first and last angiographic acquisitions. Technical success was defined as the complete absence of flow within the treated PAVM on the final angiogram. Imaging follow-up with contrast-enhanced CT was performed 3 and 12 months after the procedure and every 2 years thereafter (Figure 1f and Figure 2f). Clinical success was defined on follow-up imaging as either complete resolution of the lesion or a reduction of at least 70% in the diameter of the draining vein, without major complications [5]. If the reduction in the size of the draining vein or nidus was not significant, or if persistent enhancement of the nidus was observed on follow-up imaging, additional embolization was performed. Procedure-related complications were classified as either major or minor according to the guidelines of the Society of Interventional Radiology Standards of Practice Committee [21].

### 2.4. Statistical Analysis and AI Usage

Clinical and procedural parameters were compared between the DSA-only and CBCT groups. For categorical variables, either the chi-square test or Fisher’s exact test was used, whereas continuous variables were compared using the independent *t*-test for normally distributed data and the Mann–Whitney U test for nonparametric data. For continuous variables analyzed with the independent *t*-test, Levene’s test was used to assess the equality of variances, and Welch’s correction was applied when variances were unequal. For the primary procedural and radiation outcomes, mean differences with 95% confidence intervals and Hedges’ g effect sizes were additionally calculated. Statistical analyses were performed using SPSS software (version 25.0; IBM, Armonk, NY, USA), and statistical significance was set at *p* < 0.05. During the preparation of this work the authors used Claude Sonnet 5 (Anthropic, San Francisco, CA, USA) in order to refine the English phrasing and grammar of the manuscript.

## 3. Results

### 3.1. Patient and PAVM Characteristics

The patient and PAVM characteristics are summarized in Table 1. Of the 63 patients included in the analysis, 13 (20.6%) underwent CBCT-assisted embolization (CBCT group) and 50 (79.4%) underwent DSA-only embolization (DSA-only group). Four patients were symptomatic at presentation: three had dyspnea and one had a history of cerebral infarction. No significant differences were observed between the two groups in terms of PAVM location (*p* = 0.624), number of PAVMs per patient (1.66 ± 1.20 vs. 1.38 ± 0.87; *p* = 0.426), multiplicity (32.0% vs. 23.1%; *p* = 0.532), feeding artery diameter (4.6 ± 2.0 mm vs. 4.4 ± 1.9 mm; *p* = 0.812), or sac size (8.1 ± 4.9 mm vs. 7.2 ± 3.3 mm; *p* = 0.571). Similarly, the proportion of patients with a target AVM obscured by the mediastinum, diaphragm or adjacent pulmonary vessels did not differ significantly between the groups (34.0% vs. 53.8%, *p* = 0.214).

### 3.2. Procedure Details

Table 2 shows the procedural details for both groups. The distribution of embolic materials did not differ significantly between the groups (*p* = 0.389). Compared with the DSA-only group, the CBCT group required significantly fewer DSA acquisitions (2.4 ± 1.0 vs. 4.9 ± 2.6; mean difference 2.56, 95% CI 1.06–4.05; *p* = 0.001; Hedges’ g = 1.05), a shorter procedure time (54.6 ± 24.3 min vs. 75.1 ± 34.5 min; mean difference 20.46 min, 95% CI 3.29–37.64; *p* = 0.021; Hedges’ g = 0.61), and a reduced fluoroscopy time (14.9 ± 12.0 min vs. 25.5 ± 18.2 min; mean difference 10.64 min, 95% CI 2.04–19.25; *p* = 0.017; Hedges’ g = 0.61). DAP and cumulative air-kerma, indices of patient radiation exposure, did not differ significantly between the two groups (DAP: 87.7 ± 111.4 Gy·cm^2^ vs. 86.0 ± 58.7 Gy·cm^2^; mean difference 1.65 Gy·cm^2^, 95% CI −62.58 to 65.88; *p* = 0.959; Hedges’ g = 0.02; cumulative air-kerma: 387.2 ± 464.2 mGy vs. 354.5 ± 305.7 mGy; mean difference 32.64 mGy, 95% CI −239.77 to 305.05; *p* = 0.811; Hedges’ g = 0.07). The relative contribution of CBCT acquisition, DSA, and fluoroscopy to total radiation dose was extracted for each patient. In the CBCT group, CBCT acquisition, DSA, and fluoroscopy contributed 39.56 ± 27.00 Gy·cm^2^ (163.09 ± 140.61 mGy), 23.22 ± 15.85 Gy·cm^2^ (95.73 ± 82.53 mGy), and 23.22 ± 15.85 Gy·cm^2^ (95.73 ± 82.53 mGy) to total dose, respectively. In the DSA-only group, DSA and fluoroscopy contributed 29.80 ± 37.87 Gy·cm^2^ (131.64 ± 157.83 mGy) and 57.85 ± 73.52 Gy·cm^2^ (255.54 ± 306.38 mGy) to total dose, respectively.

### 3.3. Technical and Clinical Outcomes

Technical success was achieved in all patients in both groups (100%). No procedure-related complications were observed. Clinical success rates were 98% in the DSA-only group and 100% in the CBCT group, with no significant difference between the groups (*p* = 1.000). During follow-up, one patient in the DSA-only group developed recanalization and underwent repeat embolization, resulting in the complete exclusion of the PAVM.

## 4. Discussion

This study demonstrated that noncontrast CBCT guidance in PAVM embolization is associated with significant reductions in the number of DSA acquisitions, procedure time, and fluoroscopy time compared with the conventional DSA-only approach. Technical success was achieved in all cases, and clinical success rates were high in both groups (98% in DSA-only vs. 100% in CBCT). No adverse events were observed.

The procedural and radiation outcomes observed in this study were comparable to or exceeded those reported in previous investigations. For instance, the fluoroscopy times (14.9–25.5 min) and procedure times (54.6–75.1 min) in the CBCT group were notably shorter than those reported for fusion imaging (mean 40.6 min) [10] and were comparable to results achieved with high-frequency jet ventilation (33.4 ± 16.1 min) [22]. Although the radiation dose (mean DAP 86–88 Gy·cm^2^) was within the ranges reported in the literature, the reduction in fluoroscopy time and the need for fewer DSA acquisitions suggest enhanced procedural efficiency without increasing the radiation burden for patients or operators.

Although transcatheter embolization is the established standard for PAVMs, lesions located near the mediastinum or diaphragm often present procedural challenges due to respiratory motion and overlapping anatomical structures [7,8,9,22,23]. Traditionally, multiple DSA acquisitions at various angles are required to delineate feeding arteries, which prolongs procedure time and increases radiation exposure. To overcome these limitations, noncontrast CBCT facilitates more efficient navigation by providing a real-time 3D fluoroscopic overlay. Unlike fusion imaging, which requires time-consuming pre-procedural processing [10], this protocol significantly streamlines the workflow by allowing for direct feeder identification without the need for complex preparation. By bypassing the iterative trial-and-error process associated with traditional 2D imaging, this approach not only enhances procedural efficiency but also ensures a more predictable workflow even in challenging anatomical locations

This study has several limitations. First, the CBCT-assisted group comprised only 13 patients compared with 50 controls, which substantially limits statistical power, particularly for comparisons involving less frequent outcomes such as complications, clinical success, recurrence, and radiation exposure. Second, because of the retrospective study design, the amount of contrast media administered was not systematically recorded, despite this parameter being clinically relevant, as CBCT may reduce contrast requirements by minimizing the number of angiographic runs. Third, the selective use of CBCT at the operator’s discretion may have introduced selection bias. Fourth, the study period spanned six years, during which cumulative operator experience would be expected to increase independent of CBCT use; because CBCT was introduced later in this period, the observed reduction in procedure and fluoroscopy time may partly reflect this learning-curve effect rather than the imaging technique itself. Fifth, comparisons of procedural duration and fluoroscopy time were limited to univariate analysis; a multivariable regression adjusting for confounders such as lesion complexity, feeding artery anatomy, embolic device selection, lesion multiplicity, and location was not statistically feasible given the limited size of the CBCT group, as the number of candidate covariates would risk model overfitting. This will be addressed in a prospective study with a larger cohort, which is currently in preparation. Finally, the noncontrast CBCT overlay protocol described here was implemented on a single angiography platform; although comparable 3D overlay guidance capabilities are available on other major angiography systems, its reproducibility has not been independently validated across different platforms for PAVM embolization.

## 5. Conclusions

In conclusion, noncontrast CBCT-assisted embolization improved procedural efficiency without increasing radiation exposure. Further prospective studies with larger patient populations are needed to validate these results and better define the role of noncontrast CBCT in routine clinical practice.

## Figures and Tables

**Table 1:** Comparison of demographics and characteristics of PAVM between DSA only group and CBCT group.

	DSA Only Group (*n* = 50)	CBCT Group (*n* = 13)	*p* Value
Age (mean ± SD)	50.2 ± 12.7	55.5 ± 8.6	0.155
Sex (M:F)	6 (12.0%)/44 (88.0%)	2 (15.4%)/11 (84.6%)	0.665
Symptom (Y/N)	3 (6.0%)/47 (94.0%)	1 (7.7%)/12 (92.3%)	0.823
Location of PAVM			0.624
Right	23 (46.0%)	5 (38.5%)	
Left	16 (32.0%)	6 (46.2%)	
Both	11 (22.0%)	2 (15.4%)	
Number of PAVM (mean ± SD)	1.66 ± 1.20	1.38 ± 0.87	0.426
Multiplicity of PAVM (Y/N)	16 (32.0%)/34 (68.0%)	3 (23.1%)/10 (76.9%)	0.532
Feeding artery size (mm) (mean ± SD)	4.6 ± 2.0	4.4 ± 1.9	0.812
Sac size (mm) (mean ± SD)	8.1 ± 4.9	7.2 ± 3.3	0.571
Obscuration of target PAVM (Y/N)	17 (34.0%)	7 (53.8%)	0.214

DSA: digital subtraction angiography; CBCT: cone-beam computed tomography; SD: standard deviation; PAVM: pulmonary arteriovenous malformation.

**Table 2:** Comparison of technical factors of PAVM embolization between DSA only group and CBCT group.

	DSA Only Group (*n* = 50)	CBCT Group (*n* = 13)	*p* Value
Embolization material			0.389
Coil only	16 (32.0%)	2 (15.4%)	
Plug only	21 (42.0%)	8 (61.5%)	
Coil + Plug	13 (26.0%)	3 (23.1%)	
Number of DSA acquisitions	4.9 ± 2.6	2.4 ± 1.0	**0.001**
DAP (Gy·cm^2^) (mean ± SD)	87.7 ± 111.4	86.0 ± 58.7	0.959
Cumulative Air-Kerma (mGy) (mean ± SD)	387.2 ± 464.2	354.5 ± 305.7	0.811
Procedure time (min) (mean ± SD)	75.1 ± 34.5	54.6 ± 24.3	**0.021**
Fluoroscopy time (min) (mean ± SD)	25.5 ± 18.2	14.9 ± 12.0	**0.017**

DSA: digital subtraction angiography, CBCT: cone-beam computed tomography, DAP: dose-area product, SD: standard deviation. The bold is to indicate the clinical significance.

## Data Availability

The raw data supporting the conclusions of this article will be made available by the authors on request.

## Abbreviations

The following abbreviations are used in this manuscript:

PAVM	Pulmonary arteriovenous malformation
CBCT	Cone-beam computed tomography
DSA	Digital subtraction angiography
DAP	Dose-area product
